# Wind Turbine Blade Defect Recognition Method Based on Large-Vision-Model Transfer Learning

**DOI:** 10.3390/s25144414

**Published:** 2025-07-15

**Authors:** Xin Li, Jinghe Tian, Xinfu Pang, Li Shen, Haibo Li, Zedong Zheng

**Affiliations:** 1 Key Laboratory of Energy Saving and Controlling in Power System of Liaoning Province, Shenyang Institute of Engineering, Shenyang 110136, China; 12023201117@stu.sie.edu.cn (X.L.); pangxf@sie.edu.cn (X.P.); shenli@sie.edu.cn (L.S.); lihb@sie.edu.cn (H.L.); 2 School of Computing and Mathematical Sciences, University of Leicester, Leicester LE1 7RH, UK; zz288@leicester.ac.uk

**Keywords:** defect detection, DINOv2, Stochastic Configuration Network, wind blades, YOLOv5 network

## Abstract

Timely and accurate detection of wind turbine blade surface defects is crucial for ensuring operational safety and improving maintenance efficiency with respect to large-scale wind farms. However, existing methods often suffer from poor generalization, background interference, and inadequate real-time performance. To overcome these limitations, we developed an end-to-end defect recognition framework, structured as a three-stage process: blade localization using YOLOv5, robust feature extraction via the large vision model DINOv2, and defect classification using a Stochastic Configuration Network (SCN). Unlike conventional CNN-based approaches, the use of DINOv2 significantly improves the capability for representation under complex textures. The experimental results reveal that the proposed method achieved a classification accuracy of 97.8% and an average inference time of 19.65 ms per image, satisfying real-time requirements. Compared to traditional methods, this framework provides a more scalable, accurate, and efficient solution for the intelligent inspection and maintenance of wind turbine blades.

## 1. Introduction

### 1.1. Literature Review

As wind is a renewable energy source, wind power generation does not emit greenhouse gases or other pollutants (unlike traditional thermal power generation), thereby supporting the achievement of carbon peaking and neutralization. Severe weather and gravity-related factors will affect the long-term stable operation of wind turbine blades outdoors. Blade defects, such as paint chipping, cracking damage, and oil stains, usually develop over time. Regular inspection of wind turbine blades is currently carried out manually or with the assistance of drones. Manual detection usually requires workers to climb up tall wind turbine towers to conduct visual inspections, posing high safety risks, and this method is prone to misjudgment and missed detection. In addition, the efficiency of manual inspection is low, and it is difficult to quickly cover many wind turbines on large wind farms. Therefore, it is vital to judge blade defects safely and effectively to allow the continuous and reliable operation of a wind turbine.

In Ref. [1], the Bayesian classification method was used to classify the vibration signals generated by cracks, corrosion, loose connections, and other faults. In Ref. [2], RPCA was employed to reduce the data dimensions of vibration signals. The wind turbine blade inspection blade data set was deemed significant, and traditional machine learning was found to have low processing efficiency for large-scale data and high data quality requirements. Traditional machine learning methods must manually design and extract features, and over-reliance on prior knowledge is subject to influence by subjective factors.

In recent years, owing to advancements in deep learning advancements, neural-network-based methods have become the standard approach to wind turbine blade recognition. In Ref. [3], a characteristic map of vibration signals was input into an MCNN to extract the features of different defect types, and the ART network was used as a classifier. In Ref. [4], the Haar-AdaBoost cascade classifier was used to determine the damaged area, and then the variant VGG16 was used to classify the types of damage. However, the Haar features of this method need to be artificially designed in order to improve the model’s generalization ability. In Ref. [5], the Otsu algorithm was used to remove the complex background in blade images, and then AlexNet combined with transfer learning was used for feature extraction. Finally, a random forest was used to classify defect types. In Ref. [6], a two-stage object detection network, Faster-RCNN, with a backbone of Inception-ResNet-v2 was used to identify and classify blade defects, and the images were enhanced via flipping, brightness transformation, Gaussian blur, and other methods. In Ref. [7], the ADMM algorithm was used to reduce the weight of VGG11 for blade defect detection. In Ref. [8], the improved ResNet50 was used to replace the backbone VGG part of the SSD network to realize blade defect detection. In Ref. [9], a UAV was used to circle a wind turbine blade in order to obtain blade images, and AlexNet was used for damage classification. In Ref. [10], a multi-feature fusion residual structure named ResNet34 was proposed, with a smaller network depth than the original ResNet34. In Ref. [11], a DCNN pre-trained on ImageNet was used as a feature extractor, and an SVM was used as a classifier to classify blade defects. In Ref. [12], combined with transfer learning, ResNet101 was fine-tuned as the backbone network of an R-CNN to realize the identification of three defects: damage, cracks, and oil pollution. The improved k-means algorithm was used to reduce the influence of complex backgrounds. Although this method successfully classified these three types of defects, its accuracy still needs to be improved. In Ref. [13], VGG16 with an attention mechanism and an adaptive learning rate was used as a feature extractor. In Ref. [14], MASK-RCNN and MRNet were combined to reduce background influence. In Ref. [15], the authors compared the performance of ResNet50 and AlexNet in blade defect recognition tasks, and the results showed that ResNet50 was better. The authors of [16,17,18,19,20] all used the original or improved YOLO-series algorithms to realize defect recognition, added attention mechanisms to reduce the influence of blade image background on defect features, or used lightweight models to increase reasoning speed and lower computational power consumption. In recent years, foundation models have received extensive attention in both the language and vision domains. Large-scale language models, such as GPT and BERT, are pre-trained on vast textual corpora for language comprehension and generation. In contrast, visual foundation models such as DINOv2 and CLIP are pre-trained on large-scale image datasets to extract general-purpose visual representations. DINOv2 offers strong generalization and feature representation capabilities, particularly in complex visual environments and small-sample scenarios, making it well-suited for tasks such as wind turbine blade defect classification. In Ref. [21], a fuzzy-system-based genetic algorithm was designed to perform adaptive segmentation of trajectory sequences, enabling global optimization through dynamic mutation and crossover strategies. The core ideas of adaptive structure adjustment and optimization are relevant to visual detection in complex environments. In Ref. [22], a dual-scale complementary spatial–spectral joint model was introduced for hyperspectral image classification, improving the robustness of feature representation by integrating multi-scale spatial and spectral information. There are also recent studies offering important references for building efficient, generalizable models in the field of defect detection. In Ref. [23], an energy-efficient mechanical fault diagnosis method named SpikingFormer was proposed, inspired by neural dynamics and metric learning. This method addresses the challenge of limited sample availability in industrial scenarios by combining low-energy computing with prototype-based classification. In Ref. [24], a multi-source domain adversarial transfer network that adheres to a dual-fusion strategy was developed to enhance fault diagnosis performance in cross-domain and noisy environments. This approach integrates both feature-level and decision-level information to improve generalization. These studies reflect recent advancements in deep learning-based fault diagnosis, highlighting the importance of robustness, domain adaptation, and efficient representation learning. In Ref. [25], a meta-learning framework named MDGCML was proposed to address imbalanced open-set domain generalization in fault diagnosis. By coordinating gradients across domains and classes, it enabled balanced decision boundaries and fast adaptation to unknown conditions. This method demonstrates strong generalization in few-shot and cross-domain scenarios, offering valuable insights for robust industrial diagnosis. A summary of related work is shown in Table 1.

### 1.2. Motivation and Contributions

The analysis of vibration signals during the detection of defects in wind turbine blades is a standard method that has been applied in previous studies. However, it is hindered by environmental interference, poor real-time performance, and high equipment costs, and it is not suitable for large-scale wind turbine blade defect detection tasks. Therefore, defect detection methods based on visual images have become the mainstream. (1) Traditional machine learning requires the manual design of features, relying on domain experts’ knowledge and experience. The process of feature extraction for high-dimensional data and unstructured data is time-consuming and complex. (2) The performance of existing deep learning methods depends on image quality. Noise in the complex background of an image of a wind turbine blade may be confused with defective features of the blade. For example, background elements such as sky, clouds, and earth may look similar to blade defects, resulting in false or missed detection. (3) To ensure continuous and stable operation of a wind turbine and avoid excessively long downtimes (causing more severe damage to the blades), wind turbine blade defects should be detected in real time.

The following contributions were made to solve the above problems and improve the accuracy of wind turbine blade defect detection:

(1) An end-to-end defect detection framework for wind turbine blades was constructed, comprising three key stages—blade region localization, defect feature extraction, and defect classification—thus forming a complete visual detection process.

(2) Over-fitting suppression and robustness enhancement in blade positioning were achieved using YOLOv5. The images of wind turbine blades taken by drones show different perspectives at different angles. Changes in light will also affect the brightness or darkness (via shadows) of the blade surface, thus affecting the model’s generalizability. Therefore, we suppressed over-fitting using Mixup, brightness transformation, flipping, random scaling, and Mosaic methods for image enhancement. These augmentations improve the model’s ability to generalize across unseen scenarios, contributing to the robustness of the system. After positioning, the augmented and cropped blade regions were used as inputs for the subsequent classification process, ensuring that the DINOv2-based feature extraction and SCN-based classification operated on inputs that had already been optimized for variability and complexity, allowing the system to maintain high performance across diverse real-world conditions. This augmentation strategy effectively enhances the end-to-end robustness of the proposed defect recognition framework.

(3) We performed feature extraction of wind turbine blades based on a transfer-learning DINOv2 large vision model. In traditional feature extraction methods, features must be manually selected based on previous experience. After dimensionality reduction, some data information may be lost, resulting in limited feature expression ability. For wind turbine blade defect recognition, we propose using the DINOv2 large vision model to extract the features of the blade. This model autonomously extracts image features and possesses strong generalization capabilities, significantly enhancing the accuracy of detecting defects in turbine blades.

(4) We achieved wind turbine blade defect classification based on an SCN. The existing wind turbine blade defect detection methods have low accuracy, but using the SCN classifier can effectively improve accuracy.

The remaining parts of this study are structured as follows. The second section describes the problems inherent in wind turbine blade defect recognition and introduces the specific steps of wind turbine blade defect detection and recognition, including YOLOv5-based blade area positioning, the feature extraction of wind turbine blades via the DINOv2 large vision model, and stochastic-configuration-network-based wind turbine blade defect classification. The third section provides the details of the simulation experiment. The fourth section is a summary of our work.

## 2. Wind Turbine Blade Positioning and Defect Recognition

### 2.1. Framework for Wind Turbine Blade Defect Recognition

**Figure 1:**
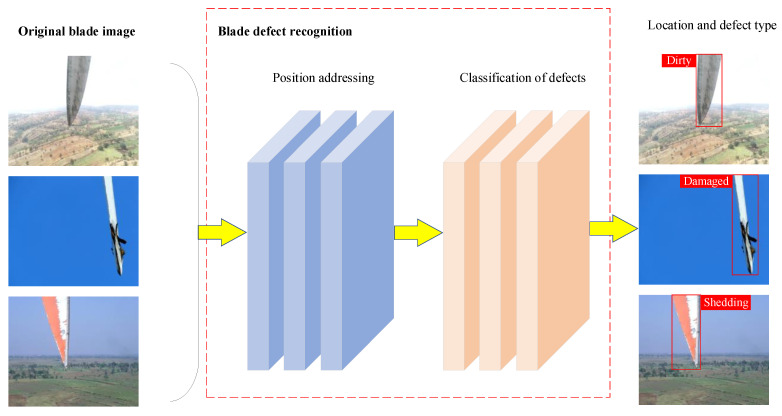
Wind turbine blade-positioning and defect classification.

Wind turbines are generally installed in windy areas such as hills, mountains, coastal regions, and vast plains. Regular maintenance of wind turbine blades is essential to ensuring the safe operation of wind turbines and prolonging their service lives. Wind turbines are generally tens of meters high. Manual blade inspection is highly risky, inefficient, and prone to subjective limitations if a turbine has been erected in a harsh natural environment. Therefore, we propose using a UAV to capture images of wind turbine blades for automatic defect detection. The process includes three parts: First, locate the blade area in the image and remove the background that does not contain the blade. Second, input the image of the blade area obtained via positioning into the defect recognition algorithm. Finally, note the position of the blade area and the corresponding defect type in the original image. This process is depicted in Figure 1.

**Figure 2:**
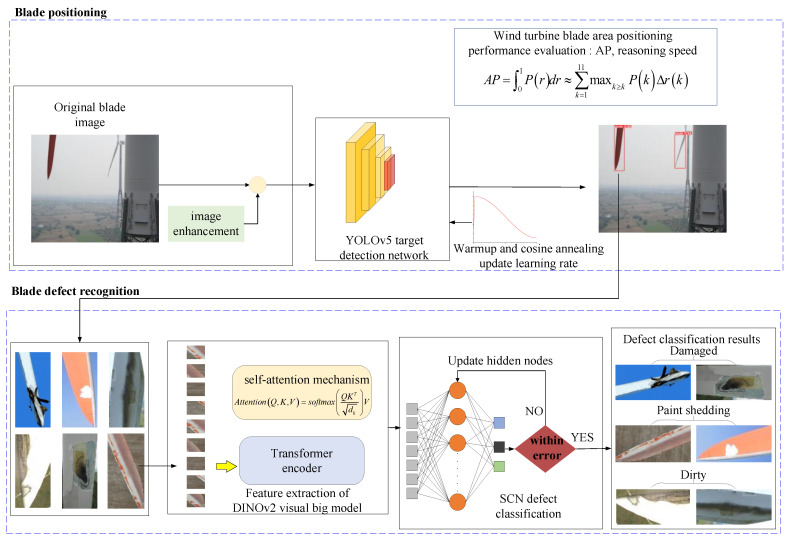
Framework of the wind turbine blade defect recognition process.

Wind turbine blade defect recognition has three parts: blade area positioning, blade feature extraction, and blade defect classification. A framework of this strategy is shown in Figure 2. (1) Wind turbine blade area positioning is determined. Original UAV-captured images containing complex backgrounds are input into the YOLOv5 object detection model. YOLOv5 accurately locates the blade regions, which are then cropped for further processing. This process effectively isolates the blade from noisy backgrounds, improving downstream feature extraction. (2) The localized blade images are resized and fed into the large vision model DINOv2, which generates a 384-dimensional feature vector representing the visual characteristics of the image. These features are robust and discriminative, and they generalize well under conditions involving complex textures. (3) The 384-dimensional feature vector extracted from the DINOv2 model is fed into a Stochastic Configuration Network for defect classification. An SCN is an incremental learning model capable of dynamically constructing its network structure while ensuring universal approximation. It selects hidden nodes based on predefined error tolerance and activation constraints, allowing for fast convergence and high classification accuracy. This process outputs the final predictions for each defect type, including damage, paint shedding, and dirt buildup.

### 2.2. Wind Turbine Blade Area Positioning Method

The next step entails locating the blade in an image and removing the complex background. The YOLOv5 detection network, known for its speed, accuracy, and simple design, is utilized to identify and localize wind turbine blade areas, aiding in efficient monitoring and analysis. The blades in the original wind turbine blade dataset image are extracted to avoid confusion between the blades’ surface defects and the background information precipitated by the feature extraction process, thereby helping the classifier improve recognition accuracy.

**Figure 3:**
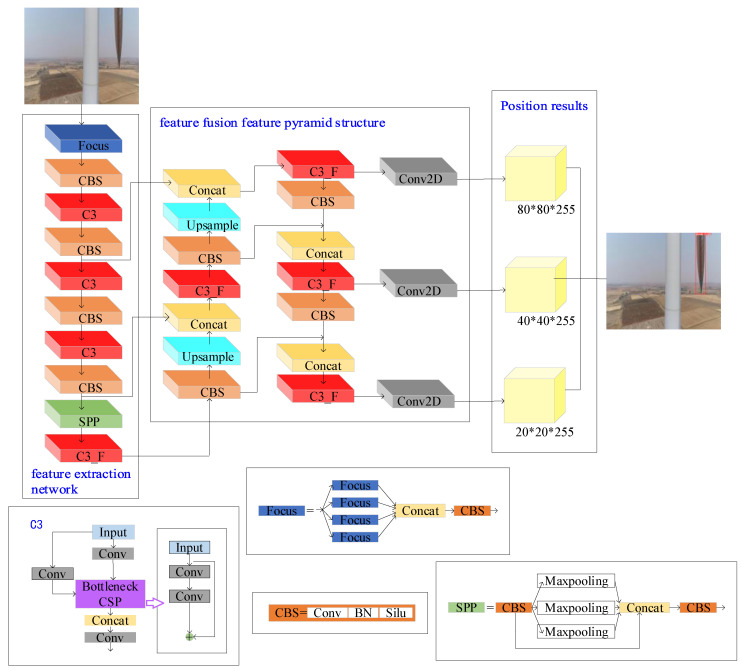
A flow chart of wind turbine blade area positioning based on YOLOv5.

YOLOv5 is a single-stage detector [26,27,28]. Owing to its high efficiency, accuracy, and ease of use, it is widely used in industrial automation, automatic driving, medical imaging, and other fields. The YOLOv5 target detection model adds a CSP structure to the backbone network based on YOLOv3 [29,30]. Its structure includes backbone feature extraction, neck feature fusion, and head target prediction. The wind turbine blade area positioning process based on YOLOv5 is shown in Figure 3.

The steps of the wind turbine blade area positioning algorithm based on YOLOv5 are as follows:

**Step 1: Wind turbine blade feature extraction.**

The original wind turbine blade image dataset—with a significant background area—captured by the UAV is input into the YOLOv5 backbone feature extraction network. Focus, CBS, C3, and SPP modules are applied to successfully extract and enhance the images.

The CBS module includes Conv (convolution), BN (batch normalization), and Silu activation functions for feature extraction. The activation function expressions are given in Equations (1) and (2), and the batch normalization process is shown in Algorithm 1. The C3 module can perform feature extraction by stacking the convolutional layers (Conv) and the BottleneckCSP module.

(1) $$Silu\left(x\right)=x\times Sigmoid(x)$$

(2) $$Sigmoid(x)=\frac{1}{1+e^{-x}}$$

**Algorithm 1:** Batch normalization**Input:** *A* = $\left\{x_{1},x_{2},x_{3,}\ldots x_{n}\right\}$, *n* is the input batch size **Output:**
*B* = $\left\{y_{1},y_{2},y_{3}\ldots y_{i}\right\}$
1 **For**
*i* = 1 to *n*
**do** 2     Calculate the input mean: $\mu_{A}=\frac{1}{n}\sum_{i=1}^{n}x_{i}$ 3     Calculate variance: $\sigma_{A}^{2}=\frac{1}{n}{\sum_{i=1}^{n}\left(x_{i}-\mu_{A}\right)}^{2}$ 4     Normalize each input using variance and mean: ${\tilde{x}}_{i}=\frac{x_{i}-\mu_{A}}{\sqrt{\sigma_{A}^{2}+\varepsilon }}$, ε is a nonzero decimal number 5     Introducing learnable parameters γ,β perform linear transformation: $y_{i}=\gamma {\hat{x}}_{i}+\beta$
6 **End For**

**Step 2: Wind turbine blade feature fusion.**

The SPP module performs maximum pooling on the feature map by pooling kernels of different sizes. Then, it performs splicing operations to obtain a new feature map, which improves the model’s adaptability to various sizes of wind turbine blades and helps express blade features. After the convolution operation, the resolution of the feature map is improved via nearest-neighbor upsampling so that this map can be fused with other feature maps to form a multi-scale feature pyramid, which can improve the detection ability of the network for different sizes of blades. The nearest neighbor upsampling is shown in Equation (3).

(3) $$x_{src}=\frac{x_{dst}Width_{scc}}{Width_{dst}},\;y_{src}=\frac{y_{dst}Height_{src}}{Height_{dst}}$$

Here, $x_{src}$ is the abscissa of a pixel in the original image, $y_{src}$ is the ordinate of a pixel in the original image, $Width_{src}$ is the width of the original image, $Height_{src}$ is the height of the original image, $x_{dst}$ is the abscissa of the corresponding pixel of the target image, $y_{dst}$ is the ordinate of the corresponding pixel of the target image, $Width_{dst}$ is the width of the target image, and $Height_{dst}$ is the height of the target image.

**Step 3: Obtain the wind turbine blade area results.**

Feature decoding is performed on the feature pyramid of the wind turbine blade neck. The head network predicts the position and size of the wind turbine blade bounding box in each image through the convolution and activation layers. It distinguishes the wind turbine blade from the background area. After upsampling, the original image size is restored, and the position of the wind turbine blade is framed.

### 2.3. Classification of Wind Turbine Blade Defects

Wind turbine blade defect classification includes feature extraction and dimension reduction with data classification. Firstly, the DINOv2 large vision model is used to extract the features of the YOLOv5-located blade images. To meet the requirements of a limited-resource environment and fast reasoning, the DINOv2 ViT-S/14 model was selected as the feature extractor. The embedding dimension of the model is 384 [31], and the obtained wind turbine blade feature vector has 384 elements. Then, the Stochastic Configuration Network is used to classify the feature vectors after data dimensionality reduction. Finally, the probability values of various defect types of wind turbine blades can be obtained.

#### 2.3.1. Method for Extracting Features Utilizing DINOv2

**Figure 4:**
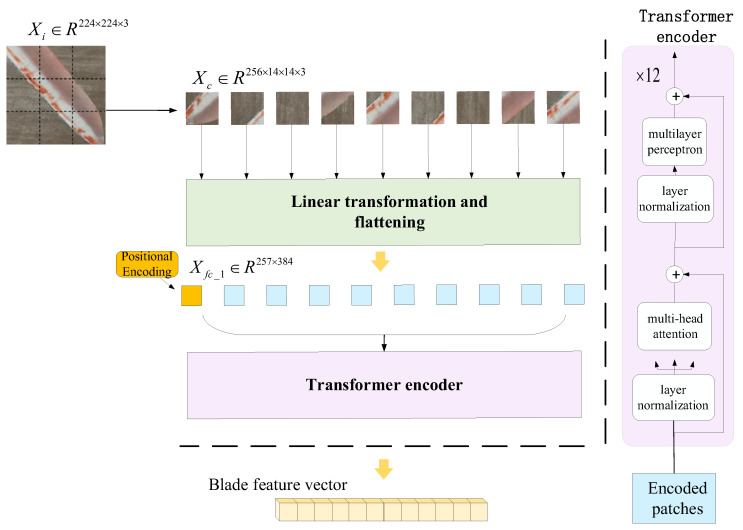
A structural diagram of the characteristics of the wind turbine blades obtained via DINOv2.

The wind turbine blade area extracted by YOLOv5 still contains a bit of background imagery. To reduce the redundant features generated by the background and the feature dimensions of the defective blade, the number of calculations carried out by the classifier is diminished, and classification efficiency is improved. Accordingly, the DINOv2ViT-S/14 model is used as the feature extractor for blade area in combination with transfer learning [31]. A flow chart of feature extraction via the DINOv2 large vision model is shown in Figure 4, and the corresponding algorithm is shown in Algorithm 2. DINOv2ViT-s/14 extracts features from unlabeled wind turbine blade image data through self-supervised learning. The student model learns the output of the teacher model and improves feature expression ability through knowledge distillation [32]. The ViT neural network has an attention mechanism that can capture the global information of an image, and parallel computing improves the running speed of the model. The attention mechanism is shown in Equation (4).

(4) $$Attention\left(Q,K,V\right)=softmax\left(\frac{QK^{T}}{\sqrt{d_{k}}}\right)V$$

In this equation, *Q* is the query matrix, *K* is the key matrix, *V* is the value matrix, and *d_k_* is the key dimension.

**Algorithm 2:** DINOv2 wind turbine blade feature extraction algorithm
**Input:** blade image $X_{i}\in {\mathbb{R}}^{224\times 224\times 3}$, $\varepsilon =10^{-6}$ **Output:** blade feature vector $y\in {\mathbb{R}}^{384}$
1 The blade image is divided into 16×16 image blocks with 14 × 14 pixels, and the wind turbine image is obtained: $X_{c}\in {\mathbb{R}}^{256\times 14\times 14\times 3}$ 2 Flatten the three-channel $X_{c}$ to get $X_{f}\in {\mathbb{R}}^{256\times 588}$ 3 $X_{f}$ is linearly projected onto a 384-dimensional vector space to obtain $X_{fc\_1}=X_{f}A^{T}+b$, $A\in {\mathbb{R}}^{384\times 588}$, $X_{fc\_1}\in {\mathbb{R}}^{256\times 384}$ 4 To distinguish the relative position of sequence elements, embedded position encoding, $X_{fc\_1}\in {\mathbb{R}}^{257\times 384}$
5 **For**
*i* = 1 to 12 **do** 6     Layer normalization: $X_{LN\_1}=\frac{X_{fc\_1}-E\left[X_{fc\_1}\right]}{\sqrt{Var[X_{fc\_1}]+\varepsilon }}\cdot \gamma +\beta$ 7     Attention mechanism: $X_{att}=Attention\left(X_{LN\_1},X_{LN\_1},X_{LN\_1}\right)$ 8     Layer normalization: $X_{LN\_2}=\frac{X_{att}-E\left[X_{att}\right]}{\sqrt{Var[X_{att}]+\varepsilon }}\cdot \gamma +\beta$ 9   The first fully connected layer of multi-layer perceptron: $X_{fc\_2}=X_{att}{A_{1}}^{T}+b$, $A_{1}\in {\mathbb{R}}^{1536\times 384}$, $X_{fc\_2}\in {\mathbb{R}}^{257\times 1536}$ 10   The second fully connected layer of multi-layer perceptron: $X_{fc\_3}=X_{fc\_2}{A_{2}}^{T}+b$, $A_{2}\in {\mathbb{R}}^{384\times 1536}$, $X_{fc\_3}\in {\mathbb{R}}^{257\times 384}$
11 **End For** 12 Layer normalization: $X_{LN\_3}=\frac{X_{fc\_3}-E\left[X_{fc\_3}\right]}{\sqrt{Var\left[X_{fc\_3}\right]+\varepsilon }}\cdot \gamma +\beta$ 13 The blade feature vector *y* is the first row element of $X_{LN\_3}$


#### 2.3.2. Feature Vector Classification Based on a Stochastic Configuration Network

**Figure 5:**
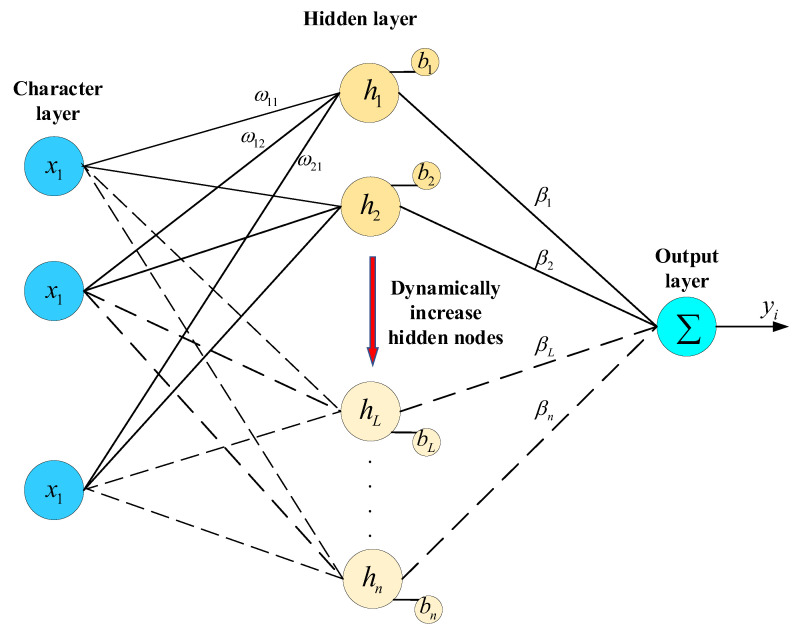
A structural diagram of the Stochastic Configuration Network.

The wind turbine blade feature vector extracted by DINOv2 has many dimensions and a large quantity of data, and it is difficult to manually distinguish the characteristics of different types of defects. Therefore, the Stochastic Configuration Network [33], which can automatically learn the classification rules from the input feature vector, is used as the classifier. It is robust and can quickly fit the data. The Stochastic Configuration Network is a random-learning algorithm with a supervision mechanism. The algorithm contains a small number of initial hidden layer nodes. According to the residual error in the input wind turbine blade feature vector fitting process and the preset tolerance error, a judgement is made regarding whether to increase the number of hidden layer nodes. Equation (7) is the current node residual calculation process. The self-monitoring mechanism randomly assigns the weights and deviations of the hidden layer, and the network structure is shown in Figure 5. In Equations (5) and (6), all the feature vectors of wind turbine blades extracted by DINOv2 are superimposed into a matrix and input into the Stochastic Configuration Network to reduce the error by automatically updating the number of hidden layer nodes until the tolerance error is satisfied. Finally, the probability values of different types of wind turbine blade defects are output.

(5) $$Y_{L-1}(x)=\sum_{j=1}^{L-1}\beta_{j}sigmoid\left(\omega_{j}^{T}x+b_{j}\right),L=1,2,3,\ldots ,n;\;f_{0}=0$$

(6) $$sigmoid\left(x\right)=\frac{1}{1-e^{-x}}$$

(7) $$e_{L-1}=f-f_{L-1}=\left[e_{L-1,1},e_{L-1,2},\ldots ,e_{L-1,n}\right]$$

Suppose the output residual error cannot meet the preset tolerance error. In that case, the number of hidden layer nodes is automatically increased. The output of the *L* hidden nodes can be expressed as Equation (8).

(8) $$h_{L}=sigmoid\left(\omega_{L}^{T}x+b_{L}\right)$$

**Theorem** **1** **[31]:** *Suppose span* $\left(\Gamma \right)$*is dense in L_2_ space, and $\forall h\in \Gamma ,0\leq \left\|h\right\|\leq b_{h}$. Given that 0<r<1 and a non-negative sequence $\{\mu_{L}\},\mu_{L}\leq 1-r$ and $\lim_{L\to +\infty }\mu_{L}=0$, for L=1,2…, denoted by Equation (9),*

(9) $$\delta_{L}=\sum_{q=1}^{m}\delta_{L,q},\delta_{L,q}=(1-r-\mu_{L}){\left\|e_{L-1}\right\|}^{2},q=1,2,\ldots ,m$$

*If h_L_ satisfies the following inequality*

(10) $${\langle e_{L-1},h_{L}\rangle }^{2}\geq {b_{h}}^{2}\delta_{L,q},q=1,2,\ldots ,m$$

*the output weights can be evaluated as follows:*

(11) $$[\beta_{1}^{*},\beta_{2}^{*},\ldots ,\beta_{L}^{*}]=\underset{\beta }{argmin}\left\|f-\sum_{j=1}^{L}\beta_{j}h_{j}(X)\right\|$$

*Then, we obtain* $\lim_{L\to +\infty }\left\|f-f_{L}^{*}\right\|=0$*, where* $f_{L}^{*}=\sum_{j=1}^{L}\beta_{j}^{*}h_{j}(X),\beta_{j}^{*}={\left[\beta_{j,1}^{*},\beta_{j,2}^{*},\ldots ,\beta_{j,m}^{*}\right]}^{T}$.

Theorem 1 demonstrates that the universal approximation capability of SCNs is theoretically guaranteed through the constraint of Inequality (10). Therefore, the continual addition of new hidden nodes based on (10) and (11) ensures that the model’s error will converge within the tolerance error.

## 3. Experimental Validation and Evaluation

### 3.1. Experimental Environment

The software and hardware equipment required for the experiment in this study are shown in Table 2.

### 3.2. Model Architecture and Parameter Setting

(1) The entire model architecture consists of three sequential modules: YOLOv5 for blade area localization, DINOv2 for visual feature extraction, and the Random Configuration Network (SCN) for defect classification. In the first stage, YOLOv5 was used for object detection. Before being input into YOLOv5, the original images captured by the unmanned aerial vehicle were adjusted to dimensions of 640 × 640, and the YOLOv5 output was used to crop the bounding box of the wind turbine blade area. In the second stage, the image of the wind turbine blade, following positioning and pruning, was resized to 224 × 224 and passed into the DINOv2 ViT-S/14 large vision model. This model output a 384-dimensional feature vector, representing the semantic and spatial information of the blade surface. In the third stage, the SCN received the DINOv2 feature vector and classified it. The SCN constructed a single-hidden layer feedforward neural network, randomly generated hidden nodes, and calculated the weights of the analytical output. The final prediction indicated whether the type of defect was damage, peeling paint, or dirt buildup. The integration of transformer-based feature extractors and adaptive SCN classifiers contributes to the overall accuracy and robustness of the proposed framework.

(2) In the blade-positioning experiment, the batch size of YOLOv5 was set to *n* = 16, the number of training instances was set to 100, the learning rate was set to η = 0.01, and the momentum was set to γ = 0.937. Choosing a lower learning rate and momentum can accelerate convergence and reduce oscillation. To accelerate the convergence of the model, improve its generalizability, and avoid premature over-fitting, Warmup and cosine annealing learning rates are used. When model training began, the Warmup preheating learning rate was selected, as shown in Equation (12). The cosine annealing strategy is shown in Equation (13).

(12) $$Warmup\_lr=lr_{in}+\left(lr_{\max}-lr_{in}\right)\cdot \frac{epoch_{now}}{epoch_{tt}}$$

(13) $$lr=\frac{1}{2}lr_{\max}\left(1+cos\left(\frac{\pi \times epoch_{now}}{epoch_{tt}}\right)\right)$$

where $lr_{in}$ is the initial learning rate of training, $lr_{\max}$ is the preset learning rate, $epoch_{now}$ is the current training round, and $epoch_{tt}$ denotes the total training rounds.

(3) In the defect type recognition experiment, the maximum number of hidden nodes ($L_{\max}$ = 500), the tolerance error (ε = 0.0001), and the maximum number of candidate nodes ($T_{\max}$ = 100) were randomly configured. Selecting 500 hidden nodes can effectively guarantee the complete convergence of the SCN, and selecting 100 maximum candidate nodes can ensure that the error is minimized every time the SCN updates hidden nodes.

### 3.3. Analysis of Wind Turbine Blade Area Positioning

**Figure 6:**
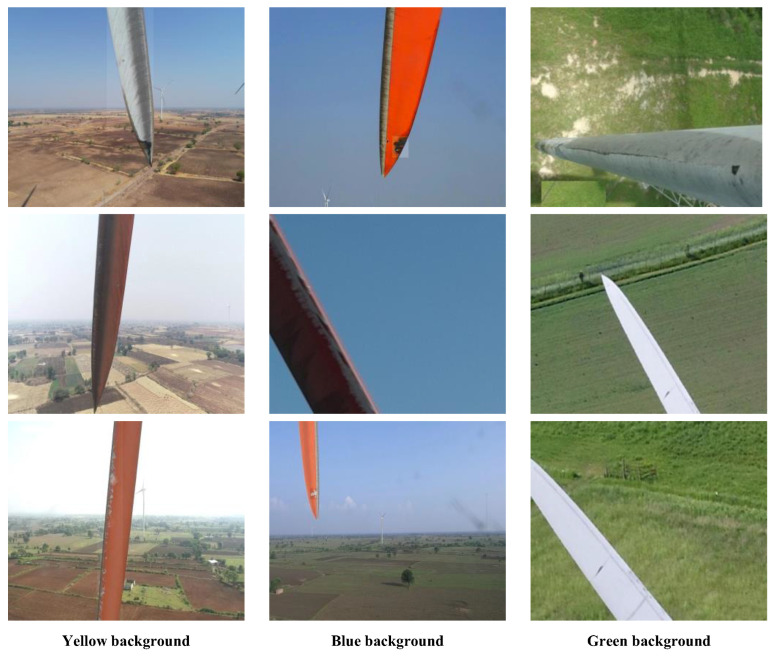
Example images from the wind turbine blade area positioning dataset.

The dataset used for blade area positioning was obtained from a public online resource. It consists of 4590 high-resolution images of wind turbine blades captured by drones under different conditions. These images cover different perspectives, lighting changes, and backgrounds, such as hills, skies, and grasslands. The dataset was randomly divided into three subsets in an 8:1:1 ratio using a Python script, with 3672 images for training, 459 images for validation, and 459 images for testing. This random division ensured the data distribution was balanced and facilitated repeatable experimental evaluation. Some examples of the blade area positioning dataset are shown in Figure 6.

**Figure 7:**
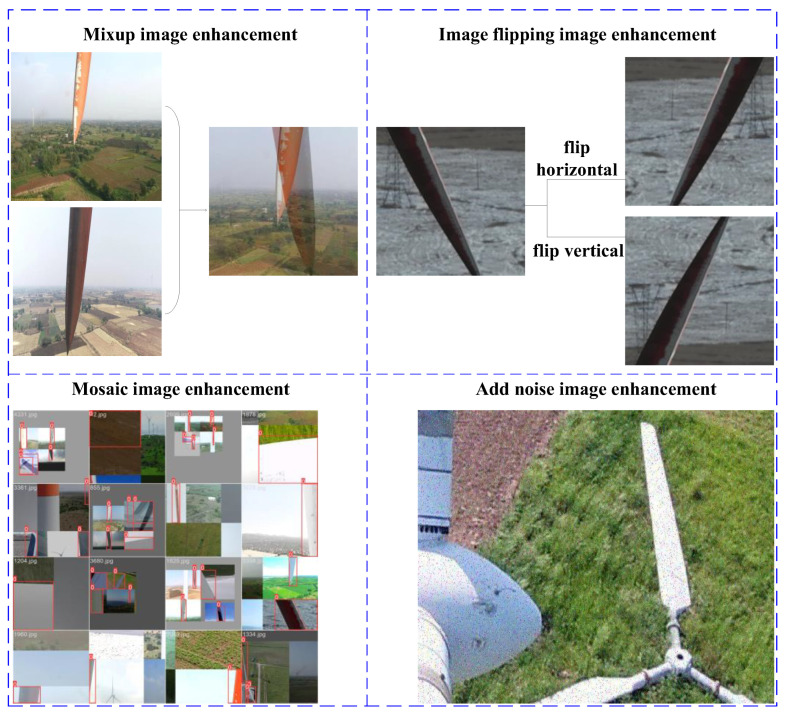
Wind turbine blade area positioning data enhancement methods.

To effectively avoid over-fitting of the YOLOv5 target detection network in the early training stage, various image data enhancement techniques were adopted to enrich the diversity and complexity of the dataset. These techniques include Mosaic enhancement, which dramatically increases the diversity of datasets by combining multiple images into one image, and the flipping are rotation of the images. These two geometric transformation techniques can simulate the wind turbine blade images taken by the UAV from different perspectives so that the model can adapt to the positioning tasks at various angles. By changing the chromaticity, the wind turbine blade images under different illumination and weather conditions were simulated by adjusting the brightness, contrast, and saturation of the images to improve the model’s robustness. By adding noise, the enhancement method simulates image noise in the natural environment, helping the model learn how to accurately detect the target in images containing noise. The image enhancement methods are shown in Figure 7.

The prediction results from the wind turbine blade area positioning experiment can be classified into four cases: instances where a positive sample was correctly classified as a positive sample, i.e., a True Positive (TP); instances where positive samples were mistakenly identified as negative samples, i.e., False Negatives (FNs); instances where negative samples were mistakenly identified as positive samples, i.e., False Positives (FPs); and instances where negative samples have been correctly identified as such, i.e., True Negatives (TNs). These four situations correspond to two evaluation indicators for evaluating the performance of wind turbine blades, namely, Precision and Recall, and the corresponding calculation equations are shown in Equations (14) and (15), respectively. Given the difficulty of direct integral calculation, we used the interpolation method to simplify the process. Specifically, it was used to calculate the average value of the accuracy rate at different recall rate levels to obtain the AP value. This index can comprehensively reflect the performance of the blade position detection network; it can be calculated as shown in Equation (16).

(14) $$precision=\frac{TP}{TP+FP}$$

(15) $$recall=\frac{TP}{TP+FN}$$

(16) $$AP=\int_{0}^{1}P\left(r\right)dr\approx \sum_{n=1}^{11}\max_{\tilde{n}\geq n}P\left(\tilde{n}\right)\Delta r\left(n\right)$$

The YOLO series single-stage target detection model and Faster-RCNN two-stage model were used to locate the wind turbine blades in this experiment. The accuracy of each model in locating wind turbine blades is shown in Table 3. AP: 50 represents the average accuracy calculated when the IOU threshold is 0.5, and the formula for calculating the IOU is shown in Equation (17). The IOUs of the predicted and actual bounding boxes were calculated. If the IOU ≥ 0.5, the prediction result was considered correct.

(17) $$IOU=\frac{area\left(B_{p}\cap B_{ab}\right)}{area\left(B_{p}\cup B_{ab}\right)}$$

In this equation, $B_{p}$ is the model’s prediction boundary, and $B_{ab}$ is the actual boundary of the wind turbine blade.

**Figure 8:**
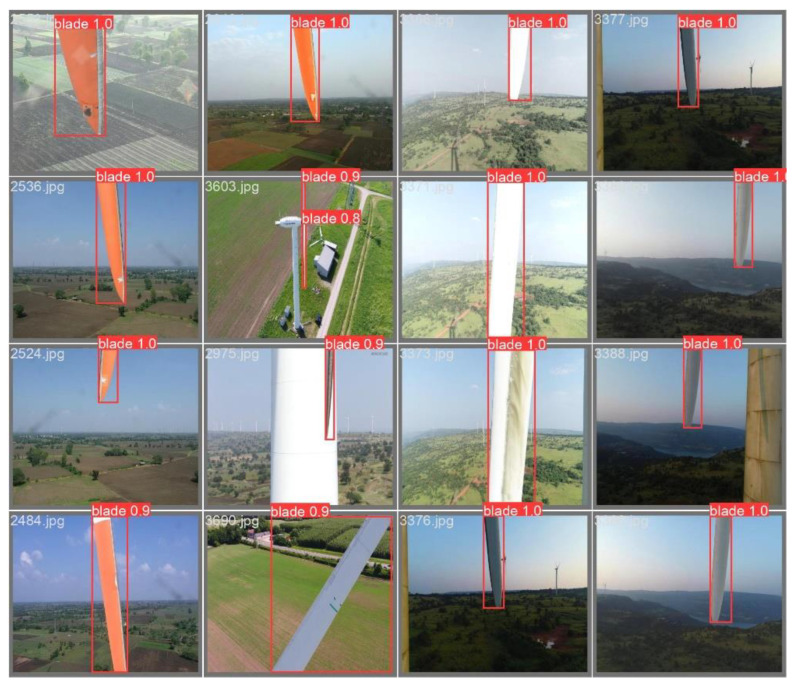
YOLOv5 wind turbine blade positioning.

As shown in Table 3, YOLOv5 had the best accuracy and speed and is the preferred model for wind turbine blade area positioning tasks. The accuracy of the FasterRCNN two-stage target detection network was much lower than that of the YOLO series, and its inference speed was also lower than that of YOLOv5 and YOLOv7. Under our experimental conditions, involving the use of an RTX4090 graphics card, YOLOv5’s speed in locating the original image of a single wind turbine blade reached 9.0 ms, meeting the real-time requirements of wind turbine inspection tasks. The experimental results regarding YOLOv5’s ability to locate wind turbine blades are shown in Figure 8.

**Figure 9:**
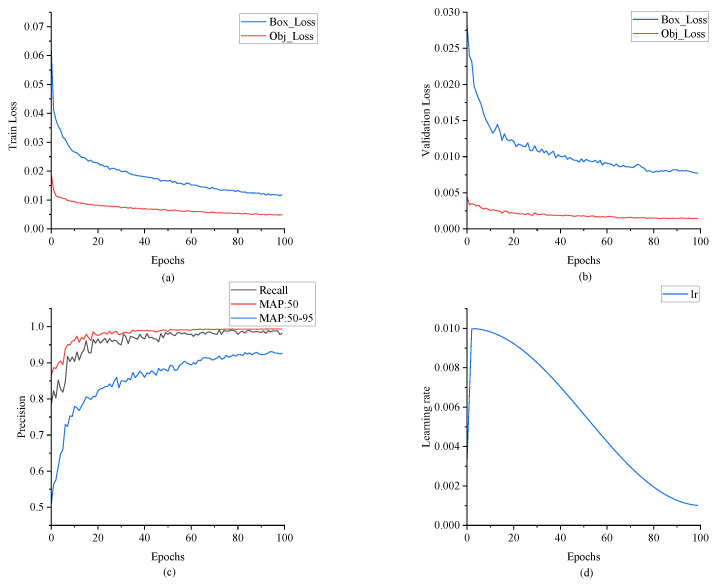
YOLOv5 training curve: (**a**) training set loss curve; (**b**) verification set loss curve; (**c**) recall, MAP:50, and MAP50-95; and (**d**) learning rate.

Figure 9a–d show the trend of the learning rate during the training process. Warmup and cosine decay strategies were adopted. After three rounds of preheating, the learning rate reached the preset value and then decayed with the cosine curve. In the Warmup rounds, a lower learning rate was used for training to avoid over-fitting and reduce the oscillation of the training process. After the model became stable, the preset learning rate was used for training. The cosine annealing strategy allows the learning rate to change according to the cosine function during the training process, allowing a smooth attenuation of the learning rate.

### 3.4. Analysis of Wind Turbine Blade Defect Classification

The dataset used to classify wind turbine blade defects consisted of the blade area images located by YOLOv5, comprising a total of 2989 images. The specific categories and division of wind turbine blade images are shown in Table 4.

**Figure 10:**
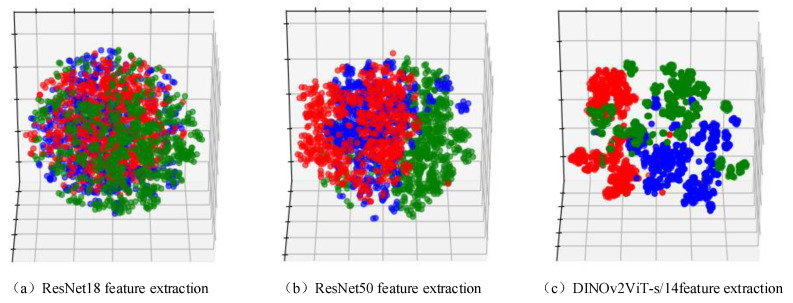
Wind turbine blade feature visualization: (**a**) ResNet18, (**b**) ResNet50, and (**c**) DINOv2ViT-s/14 feature extraction.

The feature vectors extracted by DINOv2ViT-s/14, ResNet50, and ResNet18 were reduced to three-dimensional space using the t-SNE algorithm to realize feature visualization [34]. t-SNE can reduce the dimensions of a feature vector and adapt to human observation. However, it also leads to a loss of some feature information. The feature vectors extracted by DINOv2ViT-s/14 allowed for the classification of the wind turbine defects into three categories. The vectors extracted by ResNet18 and ResNet50 are very chaotic, making the defect classification accuracy of the Stochastic Configuration Network low. Feature visualization is shown in Figure 10.

**Figure 11:**
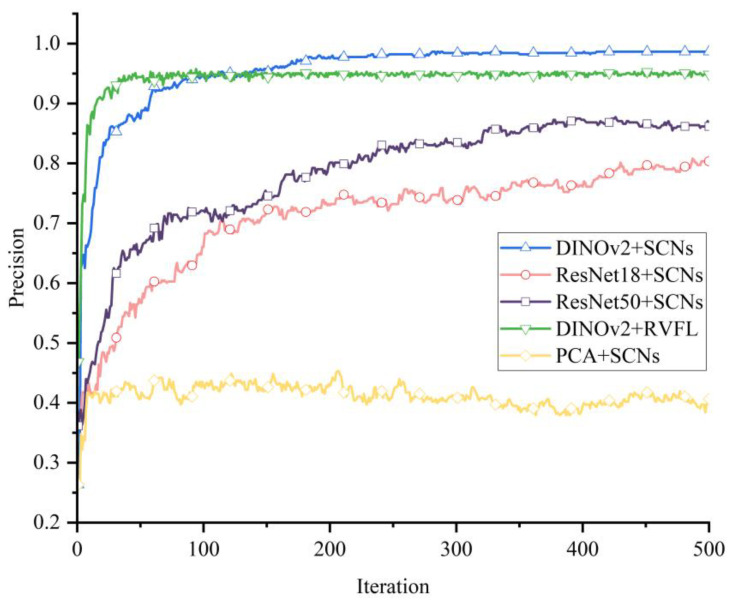
Experimental curve of defect classification.

As shown in Figure 11, using the pre-trained DINOv2ViT-s/14 model as the feature extractor and an SCN as the classifier yielded the highest wind turbine blade defect classification accuracy. The feature vector extracted by PCA had the lowest classification accuracy, mainly because PCA does not rely on prior experience and can only capture the linear features of the data, making it difficult to deal with high-dimensional data such as images. When combined with DINOv2ViT-s/14 and transfer learning, ResNet’s feature extraction effect with respect to ImageNet pre-training was better than that of PCA. Therefore, the feature extraction method consisting of applying DINOv2 and ResNet combined with transfer learning effectively improved the accuracy of the classifier.

As shown in Table 5, which compares the SCN and other classification algorithms, K-means clustering yielded a value of *k* = 3, and the KNN yielded an initial *k* = 5; SVM adopts a linear kernel function, and the evaluation standard for random forest is the Gini coefficient. The experimental results show that the SCN with automatically updated nodes and randomly assigned weights and offsets can reduce the error, effectively fit wind turbine blade data exhibiting different defect types, and improve the accuracy of defect classification. The results show that the performance of DINOv2 is significantly better than that of ResNet18 for the same classifier, reflecting the enhancement of its visual representation learning ability. When using the features extracted by DINOv2, SCN exhibited the best classification performance among all the evaluated classifiers, verifying that the SCN has stronger discriminative learning and recognition ability. The DINOv2 + SCN method exhibited good feature extraction ability and could effectively classify defective wind turbine blades.

To evaluate the feasibility of real-time deployment, the inference speed of each component in the proposed defect recognition framework was measured. The YOLOv5-based blade-positioning module achieved an average inference time of 9.0 ms per image, while the DINOv2 model required 10.60 ms for feature extraction. The SCN classifier completed defect-type classification with a time of only 0.05 ms per image. The total end-to-end inference time was approximately 19.65 ms, which corresponds to over 50 frames per second (FPS). This result demonstrates that the proposed method satisfies the requirements for real-time blade defect detection.

To evaluate the contribution of the blade region localization stage, an ablation experiment was conducted by removing the YOLOv5-based positioning module. Under these conditions, the original UAV images containing background elements such as sky, hills, and vegetation were directly input into the DINOv2 feature extractor without being cropped. As shown in Table 5, the classification accuracy dropped from 0.987 (with YOLOv5) to 0.944 (without YOLOv5). This result confirms that YOLOv5 can effectively suppress background interference and enable more focused and accurate feature extraction by DINOv2, thereby improving overall classification performance.

## 4. Conclusions

To address the low positioning efficiency for wind turbine blades, we used the YOLOv5 target detection network to remove complex background areas and retain the areas containing the blades. Aiming to address the poor robustness of feature extraction, poor adaptability to different defect types, and the low accuracy of defect classification for defective wind turbine blades, we propose a method for detecting and classifying defects in wind turbine blades based on using DINOv2ViT-s/14 as a feature extractor and a random configuration network, combined with transfer learning, as a classifier. Our conclusions are as follows.

(1) The original image data contain many complex background images, interfering with detecting blade defects. YOLOv5 was used to crop the blade areas. To avoid premature over-fitting of YOLOv5, several image enhancement techniques were applied to enhance dataset diversity. This strategy broadened data variation, improving model generalization and robustness.

(2) To address the problem wherein the traditional feature extraction method relies on prior experience, manual design, and the extraction of features, resulting in poor expression ability and low classification accuracy, we used DINOv2ViT-s/14 combined with transfer learning as a feature extractor and a random configuration network as a classifier., The accuracy of classifying defective wind turbine blades reached 98.7% using this method.

In this study, when using an Nvidia RTX4090 GPU, the AP: 50 of the wind turbine blade area was 99.4%, the inference speed for a single picture was 9.0 ms, and the wind turbine blade defect classification accuracy was 98.7%. The proposed method outperforms existing techniques in both accuracy and speed for wind turbine blade image positioning and defect classification.

While the proposed method effectively classifies the surface defects of wind turbine blades, it does not provide information about the shape or extent of the defects, limiting its ability to support finer-grained maintenance strategies that rely on a quantitative analysis of defect areas. Future work will explore the integration of segmentation networks to extract the exact locations and sizes of defects, enabling severity-level estimation and enhancing the practical value of the system in condition-based maintenance scenarios.

## Figures and Tables

**Table 1:** Summary of related work.

References	Wind Turbine Blade Positioning	Blade Defect Feature Extraction	Blade Defect Classification
[1]	-	Descriptive statistical parameters + J48 decision tree algorithm	Bayesian classification
[2]	-	RPCA	RPCA
[3]	-	MCNN	ART
[4]	Haar-AdaBoost	Improved VGG16	Fully connected layer of VGG16
[5]	Otsu algorithm	AlexNet	Random forest
[6]	-	Inception-ResNet-v2	Fully connected layer of Faster-RCNN
[7]	-	Improved VGG11	Fully connected layer of VGG11
[8]	-	ResNet50	Fully connected layer of SSD
[9]	-	AlexNet	AlexNet
[10]	-	Improved ResNet34	Fully connected layer of
[11]	-	DCNN	SVM
[12]	Improved k-means algorithm	Fine-tuned ResNet101	Fully connected layer of R-CNN
[13]	-	Improved VGG16	Fully connected layer of VGG16
[14]	MR algorithm	ResNet50	DenseNet-121
[15]	-	ResNet50	Fully connected layer
[16]	-	CSPDarknet+RepVGG	Fully connected layer of YOLOvX
[17]	-	CSPDarknet	Fully connected layer of YOLOv5
[18]	-	CSPDarknet	Fully connected layer of YOLOv5
[19]	-	MobileNetv3	Fully connected layer of YOLOv5
[20]	-	CSPDarknet	Fully connected layer
This study	YOLOv5	Feature extraction of DINOv2 large vision model	Stochastic Configuration Network

**Table 2:** Experimental equipment.

Computer Software and Hardware	Version/Model
GPU	Nvidia RTX 4090 (24 GB)
Python	3.8
CPU	Intel Xeon Platinum 8352 V
CUDA	11.3
Operating System	Ubuntu 22.04
Pytorch	1.10

**Table 3:** Localization accuracy of wind turbine blade area for each model.

Blade-Positioning Model	AP:50	Reasoning Speed
YOLOv3	0.993	10.3 ms
YOLOv5	0.994	9.0 ms
YOLOv7	0.941	8.1 ms
YOLOv8	0.990	21.9 ms
YOLOv9 FasterRCNN	0.994 0.909	31.3 ms 9.6 ms

**Table 4:** Division and statistics of wind turbine blade defect dataset.

Sample	Damage	Paint Shedding	Dirt Buildup
Training set	647	687	758
Validation set	138	148	163
Testing set	139	147	162
Total images	924	982	1083

**Table 5:** Experimental results of wind turbine blade defect classification.

Wind Turbine Blade Defect Classification Algorithm	Accuracy
DINOv2 + SCN	0.987
DINOv2 + Kmeans	0.515
DINOv2 + KNN	0.982
DINOv2 + SVM	0.906
DINOv2 + random forest	0.983
DINOv2 + RVFL	0.958
ResNet50 + SCN	0.873
ResNet18 + SCN	0.795
PCA + SCN	0.453
RESNet18 + KNN	0.759
RESNet18 + Kmeans	0.360
DINOv2 + SCN (No YOLOv5 positioning)	0.944

## Data Availability

The data that support the findings of this study are available from the corresponding author upon reasonable request.

## Abbreviations

YOLO	You Only Look Once
SCN	Stochastic Configuration Network
DINO	DETR with Improved Denoising Anchor Boxes
CNN(Conv)	Convolutional Neural Network
Faster-RCNN	Faster Region Convolutional Neural Network
VGG	Visual Geometry Group Network
ResNet	Residual Network
SSD	Single-Shot Multi-Box Detector
SVM	Support Vector Machine
RPCA	Recursive Principal Component Analysis
MCNN	Multi-Channel Convolutional Neural Network
ART	Adaptive Resonance Theory
CSP	Cross-Stage Partial Network
CBS	Conv + Batch Normalization + Silu
SPP	Spatial Pyramid Pooling
BN	Batch Normalization
Silu	Sigmoid Linear Unit
AP	Average Precision
IOU	Intersection over Union
PCA	Principal Component Analysis
t-SNE	t-Distributed Stochastic Neighbor Embedding
KNN	K Nearest Neighbor
K-means	k-Means Clustering
